# Innovating Leishmaniasis Treatment: A Critical Chemist’s Review of Inorganic Nanomaterials

**DOI:** 10.1021/acsinfecdis.4c00231

**Published:** 2024-07-13

**Authors:** Isabela A. A. Bessa, Dayenny L. D’Amato, Ana Beatriz C. Souza, Daniel P. Levita, Camille C. Mello, Aline F. M. da Silva, Thiago C. dos Santos, Célia M. Ronconi

**Affiliations:** † Departamento de Química Inorgânica, Universidade Federal Fluminense, Campus do Valonguinho, Niterói, RJ 24020-150, Brazil; ‡ Instituto de Química, Universidade Federal do Rio de Janeiro. Av. Athos da Silveira Ramos 149, CT, Cidade Universitária, Rio de Janeiro, RJ 21941-909, Brazil

**Keywords:** Leishmaniasis, neglected tropical diseases, inorganic nanomaterials, metallic nanoparticles, oxide nanoparticles, carbon-based materials, drug delivery systems

## Abstract

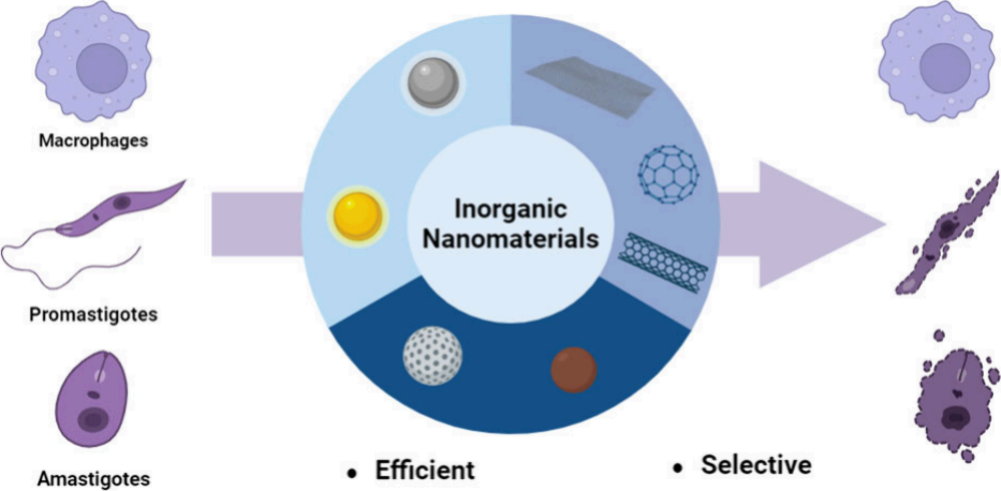

Leishmaniasis, a critical Neglected Tropical Disease caused by *Leishmania* protozoa, represents a significant global health risk, particularly in resource-limited regions. Conventional treatments are effective but suffer from serious limitations, such as toxicity, prolonged treatment courses, and rising drug resistance. Herein, we highlight the potential of inorganic nanomaterials as an innovative approach to enhance Leishmaniasis therapy, aligning with the One Health concept by considering these treatments’ environmental, veterinary, and public health impacts. By leveraging the adjustable properties of these nanomaterials—including size, shape, and surface charge, tailored treatments for various diseases can be developed that are less harmful to the environment and nontarget species. We review recent advances in metal-, oxide-, and carbon-based nanomaterials for combating Leishmaniasis, examining their mechanisms of action and their dual use as standalone treatments or drug delivery systems. Our analysis highlights a promising yet underexplored frontier in employing these materials for more holistic and effective disease management.

## INTRODUCTION

During the 73rd World Health Assembly at the end of 2020, the World Health Organization (WHO) published a document titled “Ending the Neglect to Attain The Sustainable Development Goals: A Road Map for Neglected Tropical Diseases 2021–2030”, aiming to prevent, control, eliminate, or eradicate 20 endemic diseases by the end of 2030. Since these diseases primarily affect marginalized populations in tropical and subtropical countries, they can be classified as Neglected Tropical Diseases (NTD). The Sustainable Development Goals (SDGs) explicitly include good health and well-being as the main objectives toward achieving a better world. However, challenges such as inadequate sanitation, poor living conditions, fragile health systems, limited treatment options, and inadequate vector control contribute to the persistence of NTD. Consequently, issues like poverty, hunger, and socioeconomic disparities endure over time.^1^ The socioeconomic fallout from the COVID-19 pandemic has further exacerbated extreme poverty and driven migration to rural areas, where susceptibility to infection is heightened.^2,3^ Understanding the intricate interconnectedness between human activities, animal health, and environmental factors is crucial in addressing these challenges.^4^ The One Health approach embodies this interdisciplinary perspective, recognizing the interdependence of human, animal, and environmental health. It advocates for collaborative efforts across various disciplines to develop comprehensive strategies for preventing and managing complex health threats such as the NTD. Currently, for these NTD, the estimated incidence rate is 1 billion people per year with a mortality rate of 200,000 per year.^5^

Among the NTD, Leishmaniasis is one of the main diseases compromising global health. It is caused by protozoans of the genus *Leishmania*, which includes more than 50 species transmitted by the bite of sandflies. Depending on the geographical location and incidence area, different species of *Leishmania* are found. In the Eastern Hemisphere, also known as the Old World (OW), the main species identified are *L. donovani*, *L. tropica*, *L. infantum* and *L. major*. In the Western Hemisphere, or New World (NW), the species include *L. mexicana*, *L. amazonensis* and *L. braziliensis*.^6^ These species determine the disease’s manifestation, which consists of two main clinical forms: cutaneous (CL) and visceral Leishmaniasis (VL). CL forms ulcers on skin and mucous tissues such as the throat, nose, and mouth. These ulcers, often visible, can cause stigma and bias. For mucous tissues, the only treatment available involves removal of the damaged tissue, further contributing to the stigma. VL, the most severe form of disease, affects internal organs like the liver and spleen, with the main symptoms including fever, weight loss (mostly of muscle tissues) and anemia.^7,8^ According to the WHO, there were 700,000 to 1 million new cases of CL in 2023, endemic in countries such as Afghanistan, Algeria, Brazil, Colombia, Iraq, Libya, Pakistan, Peru, Syria, and Tunisia. Meanwhile, 50,000 to 90,000 new cases of VL were registered in 2023, concentrated in Brazil, China, Ethiopia, Eritrea, India, Kenya, Somalia, South Sudan, Sudan, and Yemen.^9^

**Figure 1:**
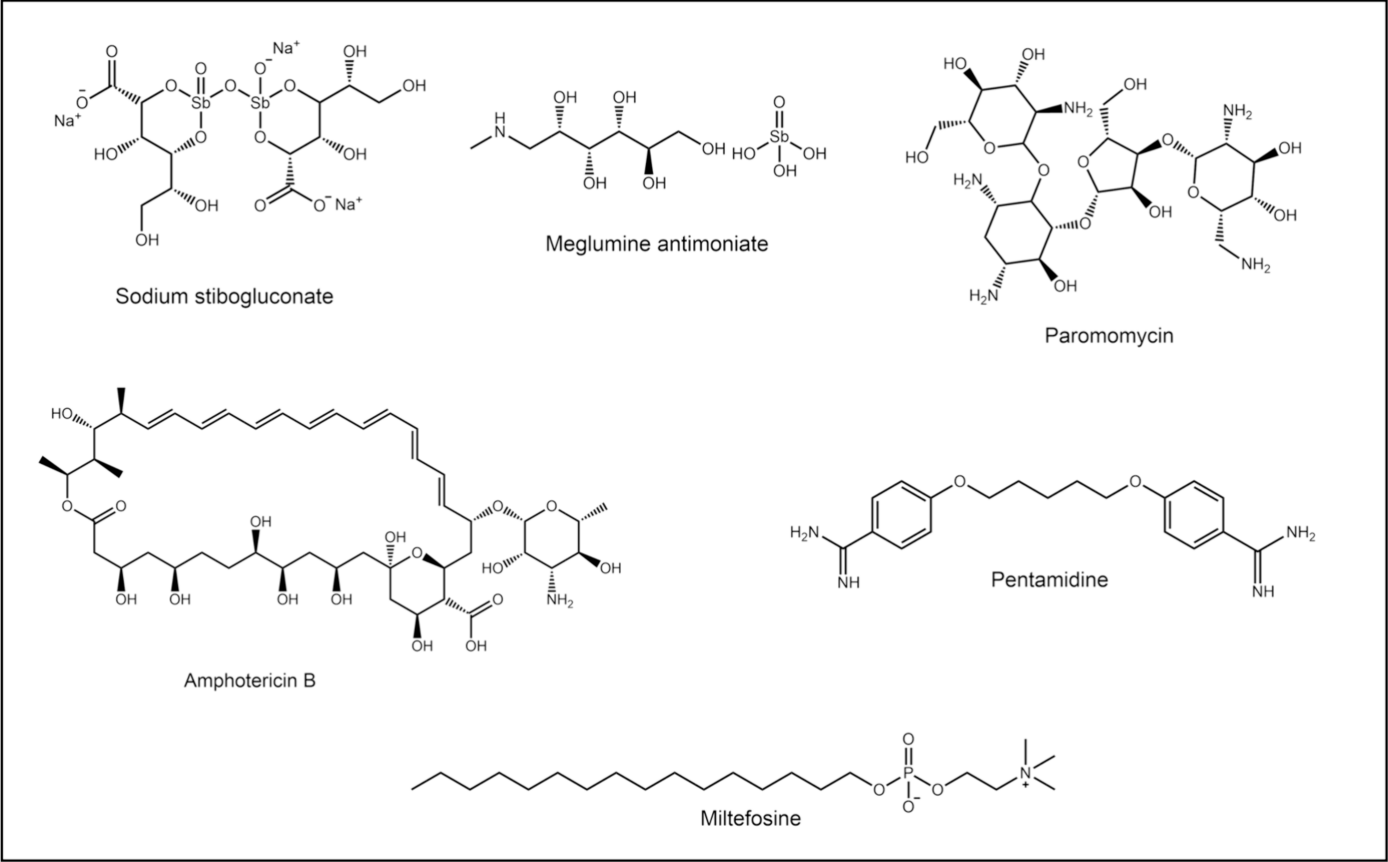
Most common drugs used in the treatment of Leishmaniasis.

Chemotherapy is the basis of the Leishmaniasis treatment. The few drugs available are poorly soluble in physiological media, making their administration, usually intravenous or intramuscular, very painful. Additionally, the several side effects caused by their administration are the main reasons for treatment abandonment, leading to an increase in drug resistance.^10^ Pentavalent antimonial-based drugs were considered the first-line treatment for many years. Although their mechanisms of action are not fully understood, it is suggested to involve the oxidation of fatty acids and adenosine diphosphate phosphorylation, leading to apoptosis.^11^ However, the need for high concentrations of Sb(V) to overcome drug resistance, along with associated toxicity, prompted the exploration of second-line drugs.^12^ These include amphotericin B (AmB), miltefosine, paromomycin (PMM) and pentamidine (Figure 1). AmB, an antifungal drug and the first choice after the pentavalent antimonials, exhibits antileishmanial activity through its interaction with the chromophore group and the ergosterol present in the protozoan’s membrane, increasing membrane permeability and leading to cell death.^13,14^ Since mammalian cells contain cholesterol, which are chemically similar to ergosterol, several side effects such as nephrotoxicity, hypokalemia, fever, headaches and anemia are associated with AmB administration.^15,16^ Miltefosine, an alquilphospholipidic anticancer drug and the only drug available for oral treatment, offers an advantage despite its critical side effects, such as hepatotoxicity, nephrotoxicity, gastrointestinal disturbances, and teratogenic.^17^ Its mechanism of action involves inhibiting the biosynthesis of phosphatidylcholine, thereby inducing apoptosis.^13,18^ Paromomycin, aminoglycoside-based drug with antibacterial and antileishmanial activity,^19,20^ has a mechanism of action that is not fully stablished, but studies suggest that it causes parasite death by inhibiting protein production due to a misreading of mRNA after binding to the ribosomal A-site, causing mitochondrial dysfunction.^19,21^ Pentamidine, an aromatic diamidine with activity against African trypanosomiasis, CL and VL, interferes with DNA synthesis and alters kinetoplast morphology.^22^ Its administration is restricted due to side effects such as cardiotoxicity, nephrotoxicity and hyperglycemia.^23,24^

**Figure 2:**
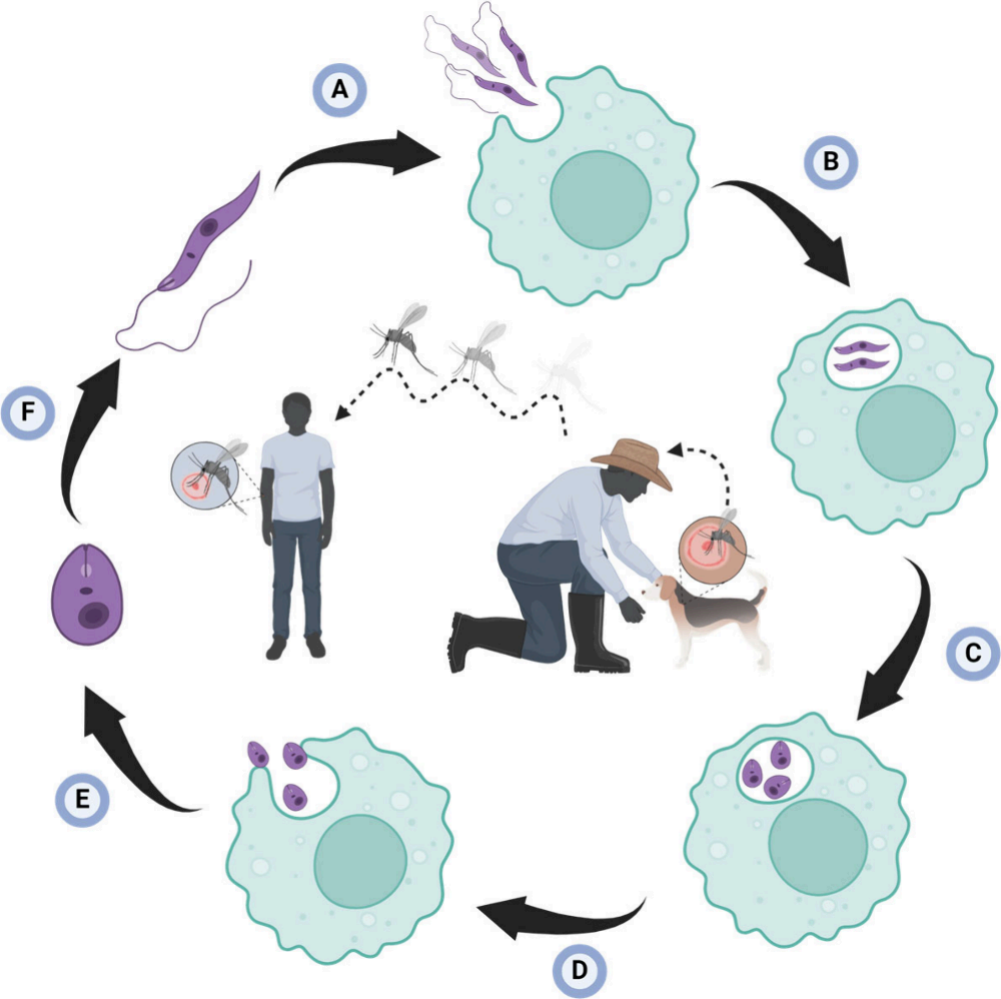
*Leishmania* parasite life cycle. (A) The promastigote form of *Leishmania* enters the mammalian host through the bite of an infected sandfly. (B) Once inside the host, the promastigotes are phagocytized by macrophage. (C) Within the macrophage, promastigotes transform into amastigotes, and (D) they reproduce within the macrophage until it bursts. (E) Subsequently, the amastigote form disseminates throughout the body, and eventually (F) they undergo differentiation back into promastigotes.

The development of new drugs is an expensive and time-consuming process, typically taking 10 to 20 years to obtain approval from federal agencies, such as US Food and Drug Administration (FDA).^25^ Moreover, the need for extensive treatments to overcome the low efficacy of current drugs and the development of drug resistance leads to environmental contamination by these drugs and their metabolites.^4,26^ Given this scenario, it is pragmatic to explore new formulations that enhance the pharmacokinetics of existing drugs, diminishing the active pharmaceutical ingredient dose at the same time. Drug delivery systems (DDS) are devices designed to protect drugs from degradation, deliver them to specifically damage tissues and release them on demand.^27^ Thus, the primary goal of this approach is to minimize side effects and improve treatment adherence. DDS are particularly promising for the treatment of Leishmaniasis, as they enable targeted and selective treatment by being engulfed by liver and spleen macrophages, where the *Leishmania* parasites reside.^28−30^ Unlikely cancer cells, which rely on passive diffusion and endocytosis mechanisms for uptake,^31,32^ macrophages can phagocytize larger foreign bodies as defensive response.^33,34^ The *Leishmania* protozoan exists in two forms: the flagellate promastigote and the nonflagellate amastigote. Transmission to a mammal occurs through a mosquito bite, with mosquito’s contaminated saliva containing the promastigote form of the parasite. Once in the bloodstream, promastigotes are phagocytized by macrophages and transformed into amastigotes, which multiply until the cell bursts and spreads throughout the body. The cycle is perpetuated when a mosquito feeds on the blood of an infected mammal. Within the mosquito’s digestive system, the protozoan differentiates back to the promastigote form (Figure 2).^35,36^

DDS can be categorized into two main groups based on their chemical nature: organic and inorganic particles. Amphiphilic organic molecules, when introduced to water, can self-assemble into structures such as micelles, vesicles, liposomes, and hydrogels. Typically, in organic DDS, the hydrophobic part of the drug interacts with the nonpolar portion of the system, while the polar part of the drug interacts with water molecules. This interaction results in a more water-soluble supramolecular system.^37,38^ For instance, AmBisome is an FDA-approved liposomal formulation of AmB that has demonstrated effectiveness and reduced side effects, although it remains costly. However, its cholesterol content restricts its use in children under one year and in elderly individuals. Additionally, there is still a moderate risk of kidney toxicity associated with its use.^10,16^

Inorganic DDS can be composed by metallic, oxides, bioceramics, and quantum dots nanoparticles.^39^ The advantage of inorganic DDS lies in the ability to control the synthesis process to achieve optimized shapes, sizes, and superficial charges,^40−42^ thereby allowing for multimodal therapy. This approach combines multiple therapeutic agents within a single structure, potentially reducing the required drug dosage and, consequently, the side effects.^28^ Furthermore, careful selection of chemical components can create stimulus-responsive DDS.^38,43,44^ For example, iron oxide superparamagnetic nanoparticles (SPIONs) can respond to an external magnetic field, leading to Neel relaxation and Brownian motion, which increases the local temperature.^45,46^ Similarly, gold nanorods (AuNRs) have a plasmonic band in the near-infrared (NIR) region that can serve as local heat source.^47^

## SCOPE AND MOTIVATION OF REVIEW

One Health is an interdisciplinary approach that recognizes the interconnection between the health of humans, animals, and our shared environment. Additionally, social, economic, ethical, and political factors should be considered in controlling complex health challenges such as zoonotic diseases, antimicrobial resistance, and environmental health issues.^26,48^ By promoting collaboration and communication among professionals from different fields, One Health aims to create a more comprehensive and effective strategy for preventing and managing health threats that span human, animal, and environmental boundaries.

In this context, DDS can significantly contribute to the One Health approach by enhancing the effectiveness and safety of treatments across humans, animals, and the environment.^49^ Outstandingly, the use of inorganic nanoparticles offers several advantages that make them invaluable for enhancing the efficacy of drug delivery systems. They can be engineered to release drugs in a controlled and sustained manner, allowing for prolonged therapeutic effects and reducing the frequency of drug administration.^50^ Additionally, they can respond to internal (*e.g.,* acid pH, hypoxia and reductor environment) or external triggers (*e.g.,* magnetic field and light exposure) to act as stimuli-responsive drug release carriers.^51^ These nanoparticles enhance the solubility of drugs, making their administration more effective. They can also be functionalized with ligands or antibodies to specifically target and accumulate at disease sites or in the intended areas of drug action. Furthermore, inorganic nanoparticles are biocompatible and well-tolerated by the human body, capable of penetrating biological barriers such as the blood-brain barrier, thus enabling the delivery of drugs to previously inaccessible body sites.^39^ Interestingly, the slightly acid intracellular environment of macrophages can be exploited as a trigger for stimuli-responsive DDS. However, these materials have been underutilized compared to organic ones for treating Leishmaniasis.^52,53^ Moreover, few studies have investigated the synergic combination of inorganic nanomaterials with drugs, especially without considering them as responsive systems.^54−62^

In this review, we present recent developments in the use of inorganic nanomaterials for Leishmaniasis treatment, categorizing them into (a) metallic nanoparticles, (b) oxides nanoparticles, and (c) carbon-based materials. Metallic and oxide nanoparticles are recognized for their ability to induce reactive oxygen species (ROS) production, which can be detrimental to the parasite. The classification of carbon-based materials, such as graphene and carbon nanotubes, is subject to debate. While some authors considered them as organic nanocarriers due to their composition of carbon atoms,^43,63^ others considered them as inorganic due to their intrinsic properties, such as absorbing NIR light.^64−66^ However, the focus of this review is not to settle this debate but rather to discuss their properties and potential applications in treatment. Thus, we aim to highlight the capabilities of inorganic nanocarriers and encourage the scientific community to integrate them into Leishmaniasis treatment efforts, conceptualizing drug delivery systems that address the needs and limitations of current treatment.

## INORGANIC MATERIALS TO TREAT LEISHMANIASIS

### Metallic Nanoparticles

**Figure 3:**
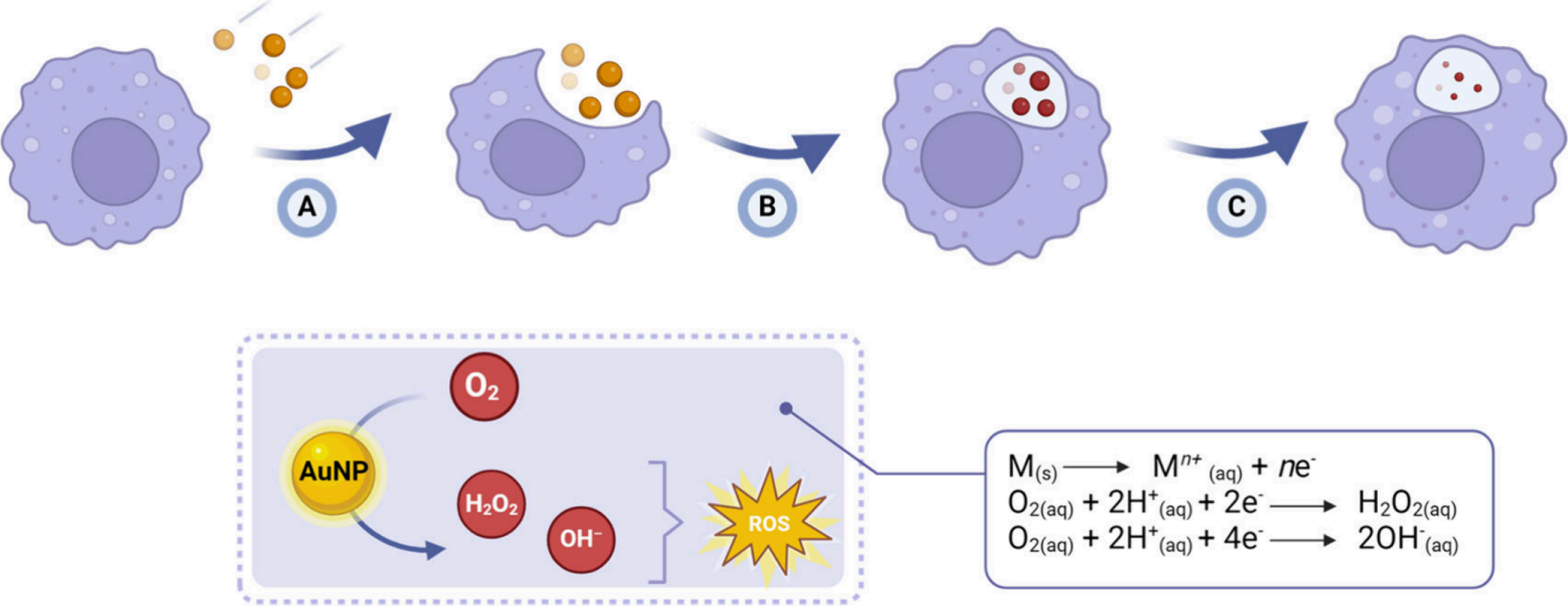
Mechanism of ROS production inside macrophages by the oxidation of metallic nanoparticles. Once in the body, the NPs (A) are recognized as foreign bodies and (B) are phagocytized by the macrophages. Inside the macrophage, (C) the NPs undergo surface oxidation (indicated by the change of color), thus producing ROS and NPs size reduction.

Metallic nanoparticles (NP) are clusters of metals in their neutral oxidation states.^67^ A key feature of this group is the presence of surface plasmons, which resonate when they interact with electromagnetic radiation, resulting in a plasmonic band. The position of the plasmonic band within the electromagnetic spectrum varies according to the nanoparticles’ shape, size, the type of metal used.^68^ The surfaces of metallic nanoparticles are easily functionalized, allowing the attachment of various molecules, such as antibodies^69^ and probes.^70^ The therapeutic applications of metallic nanoparticles have demonstrated potential in antimicrobial,^71−73^ anticancer,^74−76^ and antileishmanial activities, targeting both promastigotes and amastigotes forms of *Leishmania* protozoans.^77−82^ Their mechanism of action is primarily associated with the increased concentration of reactive oxygen species (ROS).^83,84^ Within the mammalian body, the nanoparticles are recognized as pathogens and engulfed by macrophages, which maintain an acidic environment (pH ∼ 5.8) conducive to metal oxidation.^85^ The high surface-volume ratio of metallic nanoparticles facilitates a rapid rate of surface oxidation.^86^ While macrophages produce ROS as a defense mechanism, the level of ROS generated is typically insufficient to kill *Leishmania* parasites.^87^ These parasites have developed mechanisms to survive in an oxidative environment by inhibiting the enzymatic pathways of macrophages. The release of metal ions from the nanoparticle’s lattice boosts ROS levels inside the cell, enhancing the effectiveness of the parasiticidal activity (Figure 3).^88^

The synthesis of metallic nanoparticles typically involves reducing the corresponding metal salts. Conventional chemical methods for synthesizing nanoparticles present a scalable approach, making them suitable for clinical applications. However, the high cost and toxicity of the chemicals used in these processes can restrict their use in biomedicine. Recent literature has described the preparation of metallic nanoparticles using green chemistry approach, utilizing plant extracts that serve both as reducing and capping agents.^89^ This method is cost-effective, eco-friendly,^90^ and enhances the biocompatibility of the nanoparticles by eliminating the need for toxic reduction agents, e.g., NaBH_4_ and N_2_H_4_·*x*H_2_O.^91^ Additionally, the phytochemicals in plant extracts, rich in flavonoids and polyphenolic compounds,^92^ may enhance the antileishmanial of the nanoparticles. These compounds are known for their antioxidant and anti-inflammatory properties^93^ and, although not acting as drugs themselves, can synergistically enhance the efficacy of nanoparticles against *Leishmania* protozoans by leveraging the plants’ inherent properties.^94^ However, some phytochemicals may not be as effective as conventional reducing agents, potentially leading to incomplete reduction of the metal ions.

#### Gold Nanoparticles

Gold Nanoparticles (AuNPs) are being explored as promising antileishmanial agents due to their capability to generate ROS. Also, gold compounds have been reported to inhibit Trypanothione reductase, a critical enzyme in the redox metabolism of *Leishmania.*(95,96)

Quercetin, a bioflavonoid with poor water solubility but notable antioxidant properties, shows potential effects against *Leishmania*.^97,98^ Das and co-workers synthesized quercetin-conjugated AuNP (QAuNP) and tested their efficacy against both axenic amastigotes and macrophage-infested *L. donovani* amastigotes.^99^ The inhibitory concentrations (IC_50_) for QAuNP were promising: 15 μM for axenic amastigotes and 10 μM for intracellularly infected ones. Additionally, QAuNP were effective against amastigote resistant to sodium antimony gluconate (SSG) and paromomycin (PMM), showing IC_50_ values of 40 and 35 μM against SSG-resistant strain in axenic and intramacrophage forms, respectively, and 30 and 18 μM against PMM-resistant parasites. Furthermore, QAuNP demonstrated lower cytotoxicity (CC_50_ = 1600 μM) and higher selectivity index (SI)^100^ than amphotericin B (AmB), used as positive control (IC_50_ = 0.2 μM and CC_50_ = 14 μM).

In another study, Das and co-workers developed gallic acid (GA)-functionalized AuNP (GAuNP), which, like querecetin, can induce cell death by increasing ROS levels and causing mitochondrial dysfunction.^101^ GAuNP showed significantly better antileishmanial activity than QAuNP, with IC_50_ values four times lower for axenic amastigote and three times lower for intracellular *L. donovani* amastigotes. Against SSG-resistant strains, GAuNP had an IC_50_ of nine times lower than QAuNPs but were more cytotoxic to macrophage cells (CC_50_ = 240 μM for GAuNP vs 1600 μM).

Want and co-workers explored the antileishmanial effects of pure AuNPs, with a size of 20 nm.^102^ These AuNPs showed lower antileishmanial activity compared to pentamidine, a positive control, with IC_50_ values of 18.4 μM (AuNP) vs 3.5 μM (pentamidine) for promastigote and 5.0 μM (AuNPs) vs. 1.5 μM concentration (pentamidine) was used for intramacrophage amastigote. No ROS or NO was detected, indicating that the antileishmanial effects of AuNPs were independent of ROS generation and likely resulted from their interaction with the cell membrane.

#### AuNPs Combined with Drugs

**Figure 4:**
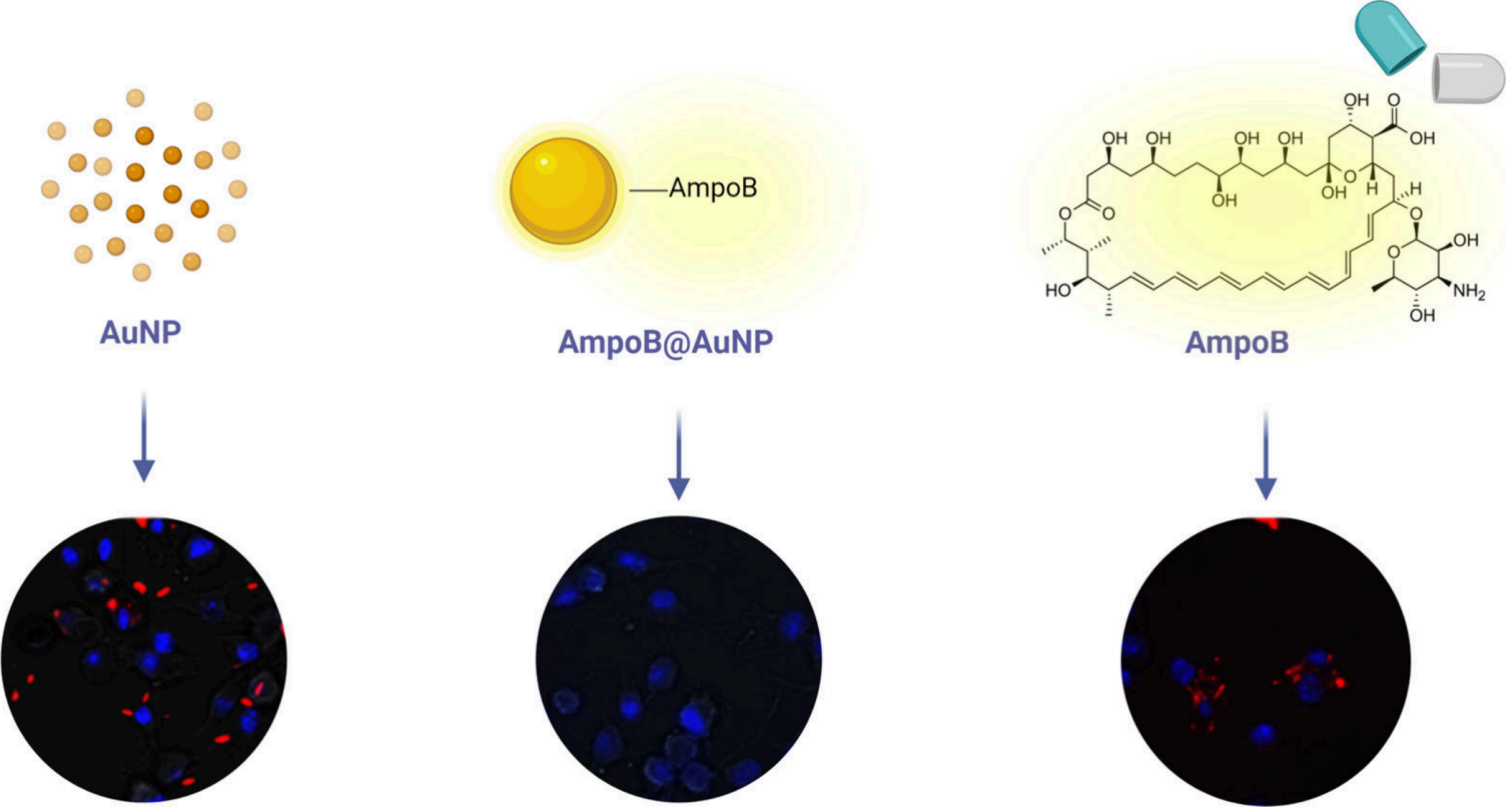
Fluorescence microscopy images of intramacrophage *L. mexicana* treated with AuNP, AmpoB@AuNP and pure AmB (AmpoB), in which the red fluorescence is related to infected macrophages, and the blue fluorescence is the nucleus. The treatment with 0.25 μg mL^–1^ AmpoB@AuNP was able to inhibit parasite infection by enabling AmB internalization. Adapted with permission from ref (54). Copyright 2021 John Wiley and Sons.

Attaching drugs to gold nanoparticles (AuNPs) can significantly enhance their water dispersibility and reduce their cytotoxicity. Ghosh and co-workers synthesized glycosylated-AuNP conjugated with AmB through an amide bond reaction (AmpoB@AuNP).^54^ The antileishmanial effectiveness of AmpoB@AuNP against *L. major* and *L. mexicana* promastigotes showed lower IC_50_ values (0.1 μg mL^–1^ for *L. major* and 0.13 μg mL^–1^ for *L. mexicana*) compared to AmB alone (0.7 μg mL^–1^ for *L. major* and 1.0 μg mL^–1^ for *L. mexicana*). Moreover, AmpoB@AuNP successfully eliminated amastigotes of both *L. major* and *L. mexicana* amastigotes (Figure 4).

AmB was also attached via amide bond to AuNP (39 nm) functionalized with lipoic acid (LP),^55^ reducing its cytotoxicity from CC_50_ of 8 μM (for AmB) to 35 μM (for AmB-LP-AuNP) in human THP-1 cells, as determined by the MTT assay. The antileishmanial activity of AmB-LP-AuNP against *L. donovani* promastigote and amastigote was notable, with an IC_50_ of 20 nM for promastigotes (compared to 50 nM for AmB) and 100 nM for amastigotes in 48 h, which is 5 times lower than that of AmB alone. Increased ROS and lipid peroxidation products indicated enhanced antileishmanial activity and reduced cytotoxicity of AmB-LP-AuNP.

Sasidharan and co-workers explored the effects of AuNP and AgNP loaded with 4′,7-dihydroxyflavone (47-DHF), an inhibitor of tyrosine aminotransferase in *L. donovani*.^103^ The cytotoxicity study of macrophage cells showed an increase in CC_50_ values from 1.13 μg mL^–1^ (for 47-DHF) to 2.95 μg mL^–1^ (for Au-47DHF) and 4.95 μg mL^–1^ (for Ag-47DHF), respectively. Ag-47DHF exhibited lower activity compared to Au-47DHF against both forms of the parasites. Both drug-loaded nanoparticles demonstrated enhanced activity against *L. donovani* compared with the pure drug. Drug release studies at pH 5.8 (mimicking the intramacrophage environment) showed that the cumulative drug release for Au-47DHF was higher than for Ag-47DHF over 72 h. The combination of low cytotoxicity to macrophage cells and improved activity against *L. donovani*, compared to the pure drug at the same concentration, positions these nanomaterials as promising drug delivery systems for Leishmaniasis treatment.

#### Silver Nanoparticles

To address the low reactivity issue of AuNPs, silver nanoparticles (AgNPs) are considered an alternative due to their higher reactivity and cost-effectiveness, as silver salts are less expensive than gold salts. Badirzadeh and co-workers explored the antileishmanial potential of curcumin-coated AgNPs against *L. major* both in vitro and in vivo.^104^ Curcumin, known for its microbiocidal, antioxidant and antitumor properties, faces clinical application challenges due to its poor water solubility, limited absorption by the human digestive system, and rapid metabolism.^105−107^ In this study, curcumin served as both a reducing and capping agent in the synthesis of AgNPs. The in vitro studies revealed that the IC_50_ for both promastigote and amastigotes forms were significantly lower than CC_50_ for macrophages. In vivo experiments demonstrated that at a concentration of 60 μg mL^–1^ (∼IC_50_), the nanoparticles reduced lesion sizes similarly to the positive control, AmB, which was used at a concentration eight times higher than the dose recommended by its manufacturer (Fungizone, 1 mg kg^–1^ for 30 days).

Mohammadi utilized ginger rhizome extract to synthesize AgNPs,^108^ observing a significant decrease in parasite propagation within 24 h at high concentrations of AgNPs, outperforming both AmB and glucantime. The AgNPs induced apoptosis in approximately 60% of promastigotes, highlighting their promise as antileishmanial agents. Zein explored the antileishmanial effects of AgNP synthesized from Eucalyptus camaldulensis leaf extract (CN-AgNPs, 12 nm) against *L. tropica.*(77) The treatment significantly reduced parasite numbers and inhibited growth by 90% after 48 h at concentration of 3.75 μg mL^–1^, outperforming the positive control, glucantime. Oliveira and co-workers also evaluated AgNPs (10 nm) obtained from *Eucalyptus grandis* leaf extract against various Leishmania species,^109^ noting a dose-dependent inhibitory effect on the growth of promastigote forms.

Biogenic silver nanoparticles (bio-AgNP) synthesized from the filamentous fungus *Fusarium oxysporum*(110) were tested against *L. amazonensis*, revealing a NO-mediated death mechanism in promastigotes and a ROS-independent mechanism in amastigotes. Moringa oleifera leaf extract was used to prepare stable AgNPs,^111^ which effectively treated ulcerative lesions in mice infected with *L. major*, showing antioxidant activity and outperforming Pentostam in efficacy.

**Figure 5:**
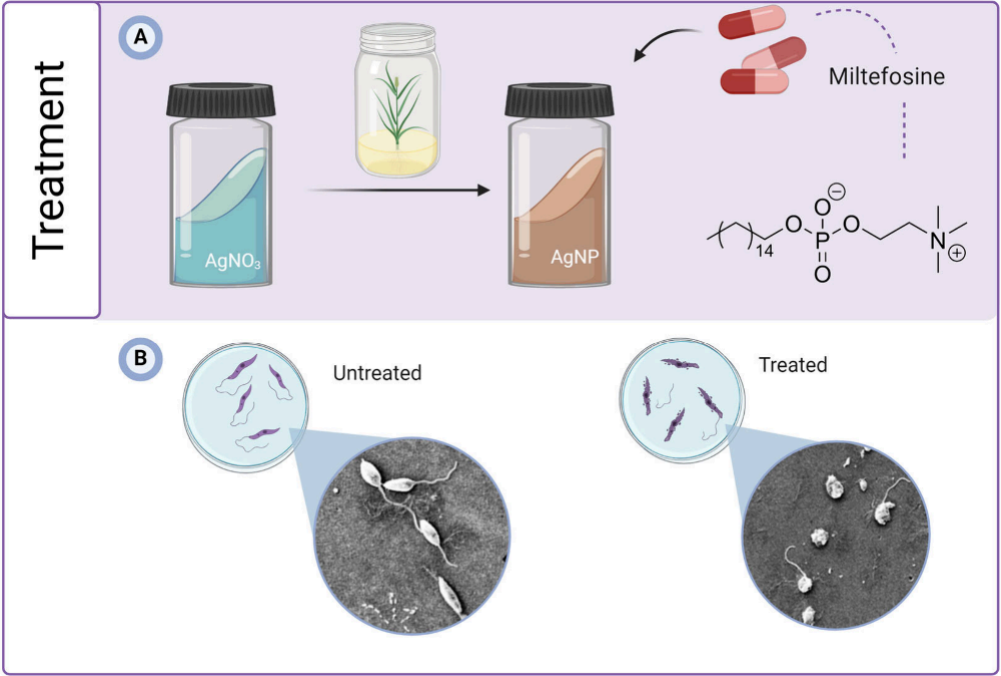
(A) Synthetic scheme of the AgNPs reduced by dill leaf extract and further combination with antileishmanial drug miltefosine. (B) SEM images showing the morphological changes in the promastigotes treated with the AgNP-miltefosine combination. Adapted with permission from ref (94). Copyright 2016 Elsevier.

While metallic nanoparticles alone exhibit antileishmanial activity through ROS generation, combining them with drugs can further enhance their effectiveness. Kalangi and co-workers used dill leaf extract to produce 35 nm AgNPs.^94^ Although these nanoparticles showed no significant antileishmanial activity alone, combining them with miltefosine increased *L. donovani* promastigote death by 33%, with SEM imaging revealing damage to the parasite’s morphology (Figure 5).

Gélvez and co-workers developed an antileishmanial nanomaterial consisting of AgNPs coated with polyvinylpyrrolidone (PVP) and meglumine antimoniate (MA) (AgNP-PVP-MA). This system could reduce *L. amazonensis* promastigote viability by 47% of without causing significant cytotoxicity to murine macrophages.^112,113^ Ahmad and co-workers created an antileishmanial agent by biosyntheszing spherical AgNP (15–20 nm) using aqueous extract of *Isatis tinctoria*, a Chinese medicinal plant.^114^ AmB was incorporated into these nanoparticles, resulting in a composite named AmB-AgNP. The antileishmanial activities of both AgNP and AmB-AgNP against *L. tropica* were assessed with and without visible light exposure. Under irradiation, Ag^+^ ions are released from the nanoparticles, promoting the production of ROS. Both materials exhibited a slight increase in antileishmanial activity upon exposure to visible light over 48 h of incubation. Specifically, maximum inhibition rates of 83% (for AgNP) and 96% (for AmB-AgNP) were observed in the presence of light compared to 73% (for AgNP) and 85% (for AmB-AgNP) in the absence of light. In contrast, the plant extract alone showed only a 43% inhibition rate after 48 h, highlighting the enhanced impact of AgNP and AmB.^115^

#### Bimetallic Nanoparticles

Interestingly, Alti and co-workers developed an Au-Ag bimetallic nanoparticle (BNP) using a reduction process with leaf extract from fenugreek, coriander, and soybean.^116^ Bimetallic nanoparticles have shown superior performance in catalytic studies and surface-enhanced Raman spectroscopy (SERS) effect, benefiting from the chemical stability of AuNP and the effective plasmonic properties of AgNP.^117,118^ In vitro studies over 48 h revealed no significant difference among Au–Ag BNP synthesized with different plant extracts, yielding IC_50_ values against *L. donovani* promastigotes as follows: 0.03 μg mL^–1^ (soybean), 0.035 μg mL^–1^ (fenugreek) and 0.035 μg mL^–1^ (coriander). Using miltefosine as a positive control, which showed an IC_50_ of 10 μg mL^–1^, the high efficiency of Au–Ag BNP was demonstrated. At concentrations up to 2.5 μg mL^–1^, the Au–Ag BNPs were not cytotoxic, causing less than 10% of macrophage death. In terms of inhibiting amastigote growth, fenugreek-derived Au–Ag BNP showed the least effect (31% of reduction) compared to soybean and coriander leaf extracts (46 and 45% of reduction, respectively).

#### Copper Nanoparticles

Copper has also gained interest for biological applications due to its pharmacological properties, including anti-inflammatory and antimicrobial effects, and its lower cost compared to gold and silver.^119−121^ Albalawi and co-workers synthesized spherical copper nanoparticles (CuNPs) ranging from 17 to 41 nm using *Capparis spinosa* fruit methanolic extract. After incorporating meglumine antimoniate (MA) into these CuNPs (MA-CuNPs), they were tested against cutaneous Leishmaniasis.^56^ The IC_50_ values for intracellular *L. major* amastigotes were 116.8 μg mL^–1^ (for CuNPs), 52.6 μg mL^–1^ (for MA), and 21.3 μg mL^–1^ (for MA-CuNPs), indicating enhanced treatment efficacy with the MA-CuNP system compared to MA alone. MA-CuNPs also decreased infection in the macrophage cells significantly. While 81.3% of macrophages were infected by untreated *L. major* promastigotes, only 5.6% were infected when pretreated with MA-CuNPs. A dose-dependent production of nitric oxide (NO) by macrophages was observed in the presence of MA-CuNPs. Topically applied MA-CuNPs on BALB/c mice infected with *L. major* fully healed the lesion within 30 days, whereas untreated mice saw an increase in the lesion size by 8.2 mm during the same period.

The described green metallic nanoparticles demonstrate promising antileishmanial activities, even against drug-resistant *Leishmania* strains, likely due to ROS production influenced by plant-derived reducing and capping agents. Indeed, the use of a plant-based approach can be a powerful tool in the context of One Health. By integrating the knowledge of pharmacists, chemists, biologists and biomedical professionals, the need of hazardous chemicals can be reduced, thus improving environmental friendliness.^122,123^ However, the bioreduction of metal salts presents a reproducibility challenge for nanoparticle production due to their heterogeneous and variable compositions. Notably, there is a scarcity of studies combining antileishmanial drugs with metallic nanoparticles to form DDS. While some research has covalently bonded drugs onto the surface of metallic nanoparticles, these cannot be strictly classified as DDS. DDS represents a more appealing strategy to enhance drug availability, dispersibility, and selectivity while preserving the drug’s molecular structure. In the case of metal-based DDS, drugs can be incorporated through supramolecular interactions onto NPs’ surface, enhancing bioavailability due to improved solubility and stability. They can also be easily modified with antibodies to target specific cells, tissues or organs, thereby increasing drug efficacy and safety.^124^ Additionally, DDS can be designed as responsive systems to release the drug in a sustained manner, ensuring optimal drug concentration available in the organism.^125^

### Metallic and Nonmetallic Oxide Nanoparticles

A variety of metallic oxide nanoparticles (MO-NPs) such as ZnO, TiO_2_, CaO, AgO, Co_3_O_4_ and MgO, are being considered as alternative treatment for infectious diseases. Their surface composition, charge, area, and small dimensions allow them to penetrate cells and disrupt the DNA and enzymes of the infectious agents, making them particularly interesting for treating Leishmaniasis.^126−131^ These MO-NPs exhibit antimicrobial properties with fewer side effects and lower toxicity to humans compared to traditional drugs.^132^

#### Metallic Oxide Nanoparticles

**Figure 6:**
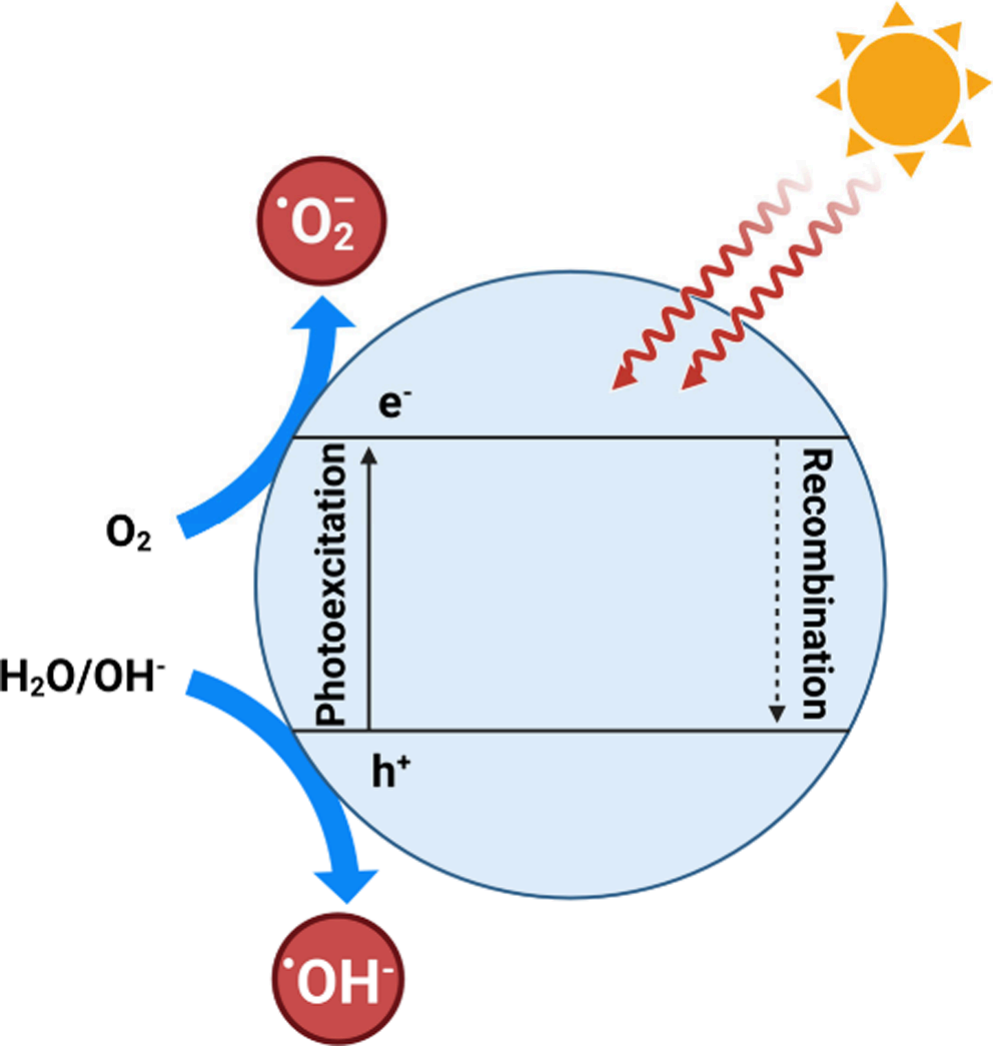
Mechanisms to generate ROS by light irradiation of the MO-NPs.

Metallic oxide nanoparticles (MO-NPs), especially those formed by transition metals ions with unfilled d-shells, facilitate electronic transitions, providing unique electronic properties.^133^ By altering their size, morphology, crystalline phase, and doping with other atoms, their bandgap energies can be modulated. Upon absorbing radiation energy exceeding their bandgap, electrons are promoted from the valence band to the conduction band, creating a positive hole (h^+^) in the valence band and enabling both the ejected electron (e^–^) and the positive hole to exhibit high reducing and oxidizing powers, respectively (Figure 6).^134−136^ This process also allows MO-NPs to generate reactive oxygen species (ROS), contributing to their application in the biomedical field.

This approach can be very interesting when considering other mammalian hosts beside humans. By engineering the band gap of these semiconductors, their photocatalytic activity can be optimized to generate ROS when exposed to solar radiation.^137^ This process can effectively target and kill the *Leishmania* parasites within the host without requiring direct or prolonged contact with the infected animals. Such an approach not only reduces the stress and handling of the animals but also enhances the efficiency and feasibility of large-scale treatment programs, making it a highly advantageous method in the fight against Leishmaniasis. Treating animals and fostering a better relationship with them aligns perfectly with the One Health concept.^138^

##### Zinc Oxide Nanoparticles and Metal-Doped Zinc Oxide

Notably in Leishmaniasis treatment where zinc oxide (ZnO) and titanium dioxide (TiO_2_) are extensively investigated for their antimicrobial activity.^136^ These nanoparticles are chemically stable, biocompatible, used in sunscreens as physical blockers,^139^ and generally recognized as safe and effective (GRASE) by the US FDA,^140−142^ making them particularly suitable for CL treatment.

Zinc oxide nanoparticles (ZnO NPs) possess a hexagonal wurtzite structure and a wide bandgap (E_g_ = 3.37 eV) that corresponds to high excitation energy in the UV region, allowing them to generate ROS in the presence of UV light.^136^ However, the potential harmful effects of UV light have limited their applications, leading to research into doping ZnO to shift its excitation energy into the visible light region for safer application.

Various studies have explored the antileishmanial potential of MO-NPs. Mahmoudi and co-workers developed a chitosan/ZnO nanocomposite with significant antileishmanial responses, demonstrating better efficiency against amastigotes than promastigotes of *L. major*.^143^ Chitosan, a natural polysaccharide, stabilizes nanomaterials in physiological environmental, enhancing their selectivity and preventing enzymatic degradation.^144^ The IC_50_ of the chitosan/ZnO nanocomposite against amastigotes, was 10 μg mL^–1^ after 72 h of incubation, five times more effective than against promastigotes. Comparably, at 200 μg mL^–1^, Chitosan/ZnO was as effective as AmB against promastigotes. The researches attributed the unexpected efficacy to potentially toxic effects of excess Zn^2+^ ions, which could inhibit the absorption of iron and copper ions. The material exhibited some promising in vitro indicators, but detailed information about the stability of the NPs is needed to further optimize the toxicity aspects before in vivo studies.

Khatami and co-workers synthesized rectangular ZnO nanoparticles (50 nm) using Stevia extract,^145^ observing decreased viability of promastigotes with increasing ZnO concentration, with an IC_50_ value between 75 and 100 μg mL^–1^. Similarly, Khan and co-workers used *Monotheca buxifolia* extract to synthesize spherical ZnO NPs (45.8 nm),^146^ noting IC_50_ values of 248 μg mL^–1^ and 251 μg mL^–1^ against *L. tropica*’s promastigote and amastigote forms. The *Monotheca buxifolia* species have several biological properties, such as vermicide, laxative and is used in the treatment of gastrointestinal disorders.^147−149^ The shape of the nanoparticles–rectangular vs spherical–may account for the variation in IC_50_ values, although further studies using consistent extracts and *Leishmania* strains are needed for confirmation.

Khashan and co-workers produced Al-doped ZnO via laser ablation in deionized water, demonstrating notable inhibition of *L. tropica* and *L. donovani* compared to nondoped ZnO nanoparticles.^140^ Al atoms were used as a nontoxic doping agent to improve ZnO’s activity, with the growth inhibition being dependent on the Al-doping ratio. By adjustment of the number of laser pulses, they synthesized five batches of ZnO and four batches of Al-doped ZnO NPs, with doping ratios ranging from 0.20 to 0.42%. Interestingly, the bandgap energies of ZnO NPs (E_g_ = 3.05–3.25 eV) and Al-doped ZnO NPs (E_g_ = 3.15–3.35 eV) showed no significant differences. The effectiveness in inhibition was Al-doping ratio dependent, suggesting that doping is a promising strategy to enhance the nanoparticles’ biological activity. However, the specific reasons why aluminum doping leads to improved biological effects have not been discussed by the authors.

Barbosa and co-workers developed nanocomposites of Ag-doped ZnO and AgO NPs (Ag-ZnO/AgO) and explored their effect on the promastigote forms of *L. braziliensis*, as well as their immunomodulatory activities.^150^ The concentration of AgO within the nanocomposites was varied, with respective compositions being 49%, 65% and 68% for materials designated as ZnO:5Ag, ZnO:9Ag and ZnO:11Ag, respectively. This increase in AgO content led to a significant improvement in the selective index (SI) against *L. braziliensis* promastigote form, with SI rising from 1.33 (for pure ZnO, IC_50_ = 928 μg mL^–1^) to 3.35 (ZnO:5Ag, IC_50_ = 397 μg mL^–1^), 52.03 (ZnO:9Ag, IC_50_ = 7.93 μg mL^–1^) and 20.38 (ZnO:11Ag, IC_50_ = 15.33 μg mL^–1^). At a concentration of 50 μg mL^–1^, the nanocomposites achieved similar effectiveness to AmB (2 μg mL^–1^) against *L. braziliensis* intramacrophage amastigote. Furthermore, these nanocomposites were observed to increase the expression of TNFR1 and TNFR2 receptors, which are known to stimulate immune cell responses. At lower concentrations of 6.25 and 12.5 μg mL^–1^ for ZnO:9Ag and ZnO:11Ag, there was a noted increase in the production of inflammatory cytokines, such as TNF-α and NO, underscoring the significant influence of the AgO content on the biological effects observed.

Cao and co-workers explored the synthesis of K-doped ZnO nanoparticles (50 nm) using a methanolic extraction of *Artemisia annua*, known for its antiparasitic effects.^151^ This plant is used in treating fevers and parasitic infections and has demonstrated antibacterial, antifungal, and anti-SARS-CoV-2 effects.^152,153^ The K-doped ZnO nanoparticles and meglumine antimoniate exhibited a similar effect on the viability of *L. tropica* promastigotes, with an IC_50_ of ca. 500 μg mL^–1^. Notably, K-doped ZnO nanoparticles were less toxic to macrophages than meglumine antimoniate, suggesting a potential advantage for their use. The authors hypothesized that the presence of K^+^ ions might disrupt the ion concentration across cell membranes, enhancing the permeability of ZnO into the cells.

MO-NPs have demonstrated significant antileishmanial activity and low toxicity to macrophages. Nonetheless, the relatively high IC_50_ values reported could potentially be decreased by leveraging the unique optical and magnetic properties of these materials. Semiconductor nanomaterials can generate ROS when exposed to light with photoenergy exceeding their bandgap. This process can lead to oxidative stress, damaging organic macromolecules and affecting nuclear, ultimately resulting in cell death.^136,140,154,155^

Nazir and co-workers conducted several studies using ZnO-based nanomaterials for photodynamic therapy (PDT) against *Leishmania*.^137,156−159^ They evaluated PEG-functionalized Ag-doped ZnO NPs (20–50 nm), with varying levels of silver content (0.5, 1, 3, 5, 7, and 9 mol % of Ag) against promastigote *L. tropica*.^137^ All materials exhibited an energy excitation around 3.2 eV and the IC_50_ value for the Ag-doped ZnO NPs ranged from 0.009 to 0.02 μg mL^–1^, significantly lower than that for the nondoped ZnO NPs (0.1 μg mL^–1^). Both the doped and undoped NPs were more effective than the positive control AmB (IC_50_ = 0.34 μg mL^–1^). To assess the effect of sunlight on parasite inhibition, cultures of intramacrophage amastigotes treated with these NPs (0.1 μg mL^–1^) were exposed to sunlight for 15 min. The Ag-doped ZnO NPs (9 mol % of Ag) managed to kill nearly 90% of the parasites, while the nondoped NPs achieved only 38% kill rate, underscoring the critical role of sunlight irradiation in ROS generation.

In subsequent research, the authors produced ZnCuO nanostructures, by doping ZnO with Cu^2+^ and combined Cu^2+^/N to adjust the conduction band and reduce the bandgap, thereby enhancing the visible light response and ROS production.^156^ Various doping ratios were tested, resulting in a decrease in bandgap energy and a dopant concentration-dependent cytotoxicity observed only at concentrations above 200 μg mL^–1^. ZnCuO3 showed the lowest IC_50_ value, consistent with an increased ROS generation. However, an overabundance of surface defects, despite initially promoting ROS production, ultimately reduced ROS levels in certain samples.

More recently, C-, N- and combined C/N-doped ZnO referred to as PC1–PC4 (representing ZnO, ZnO:N, ZnO:C, ZnO:C:N, respectively) were synthesized with sizes of 6.9, 9.8, 7.4, and 6.9 nm, respectively.^158^ The in vitro antileishmanial activity against promastigote forms of *L. tropica* demonstrated time- and dose-dependent inhibition. Notably, PC2 emerged as the most effective material, with an IC_50_ of 0.012 μg mL^–1^. This efficacy was attributed to an increased production of electron and hole pairs, leading to an enhanced ROS generation. Additionally, in vitro toxicity studies using brine shrimp indicated lower toxicity and higher viability for macrophages treated with PC2 and PC4, suggesting that N-doping offers better biocompatibility compared to C-doping. Consequently, PC2 was administered to male BALB/c mice through both intraperitoneal and topical routes to assess its potential in treating cutaneous and subcutaneous Leishmaniasis. The most favorable outcomes were observed with topical administration, where no toxicity was detected at any of the doses used, further confirming that N-doped is more biocompatible than nondoped ZnO.

##### Titanium Dioxide and Metal-Doped Titanium Dioxide Nanoparticles

Titanium dioxide nanoparticles (TiO_2_ NPs) exhibit two tetragonal crystalline phases (anatase and rutile) and one orthorhombic (brookite), with a bandgap E_g_ = 3.20 eV.^160^ Among these, the anatase phase is particularly valued for its larger surface area and higher concentration of oxygen vacancies. These characteristics provide more active sites and improve charge separation efficiency, respectively.^161^

Dolat and co-workers explored the antileishmanial effectiveness of anatase and rutile forms of TiO_2_ (32 and 51 nm, respectively) against promastigote forms of *L. major*, both with and without the presence of UVA and UVB light, irradiating the samples for 30 and 60 min.^162^ Without UV irradiation, the anatase phase of TiO_2_, at a concentration of 600 μg mL^–1^, reduced promastigote viability to 30% after 24 h. In contrast, the same level of viability reduction for the rutile phase required 48 h at the same concentration. This difference is likely due to the anatase phase’s larger surface area (200 m^2^ g^–1^) than rutile (2.5 m^2^ g^–1^), which facilitates the ROS production in higher quantities. When treated solely with radiation, UVB irradiation led to a more significant reduction in viability than UVA after 24 h.

It is important to note that UV light can be harmful to normal tissues, with potential genotoxic and mutagenic effects.^163^ To make TiO_2_ therapy safer for humans, one approach is to shift its absorption to visible light by doping TiO_2_ structure with cations.^164^ Allahverdiyev and co-workers synthesized Ag-doped TiO_2_ (TiO_2_@Ag) nanoparticles (40 nm) to assess their antileishmanial efficacy against *L. tropica* and *L. infantum* under both dark and visible light conditions.^154^ This method takes advantage of the antimicrobial activity enhancement provided by the incorporation of Ag^+^.At concentration of 25 μg mL^–1^ and with visible light irradiation, the inhibition of amastigote form of *L. tropica* and *L. infantum* was ca. as effective as in darkness.

In an effort to improve the selective index, i.e., increase the CC_50_ and/or decrease the IC_50_, *Nigella sativa* (NS) oil was used alongside TiO_2_@Ag to create a formulation that is both safe and effective against *L. tropica* promastigotes.^165^ Typically, combined therapy involves simply mixing the individual components, which may result in losing some DDS features, such as controlled drug release and enhanced drug solubility. However, this approach can reduce the toxicity and produce synergistic effects. The primary constituent of *N. sativa* oil, thymoquinone, has shown significant inhibitory effect on *L. infantum* and *L. tropica* parasites. The combined treatment demonstrated a synergic effect at concentrations of 15 μg mL^–1^ of TiO_2_@Ag + 30 μg mL^–1^ of NS, leading to a 63% reduction in promastigotes and 20 times decrease in macrophage infection with amastigotes compared to the control group. In further studies, the researches replaced the NS oil with meglumine antimoniate (MA), also in combination with TiO_2_@Ag, directly in the culture medium.^166^ This combination successfully inhibited promastigote proliferation (90% inhibition) and nearly completely inhibited intramacrophage amastigotes, also significantly reducing their metabolism and size and parasite burden of lesions in BALB/c mice. The outcomes of the combined formulation surpassed those of for pure MA or TiO_2_@Ag in all aspects. Despite these promising findings, the mechanism underlying the synergistic interactions in both combinations remain unclear. Once more, combining NPs with a known drug through simple mixing led to a loss of certain DDS advantages.

Another aspect to consider is that merely lowering the energy bandgap is not sufficient; understanding the underlying chemical phenomena is equally critical. In this context, TiO_2_ nanostructures (20 nm) doped with Fe^3+^-, Zn^2+^-, and Pt^4+^- were synthesized to decrease the bandgap energy values compared to nondoped TiO_2_, thereby potentially enhancing antileishmanial activity.^164^ The predominant phase in which these materials crystallized was anatase, which exhibited lower E_g_, values than nondoped TiO_2_: 1.88 eV for Fe^3+^-doped, 2.50 eV for Zn^2+^-doped, 3.01 eV for Pt^4+^-doped, and 3.11 eV for nondoped. At a concentration of 25 μg mL^–1^, these materials were exposed to a culture of *L. amazonensis* amastigote and irradiated for 40 min using an LED laser as the visible light source, which had two main emissions at 450 and 552 nm. Only the photoactivated Pt^4+^-doped (IC_50_ = 18.2 μg mL^–1^, SI = 3.2) and Zn^2+^-doped TiO_2_ NPs (IC_50_ = 16.4 μg mL^–1^, SI = 1.9) exhibited activity against the amastigote forms. Intriguingly, Fe^3+^-doped TiO_2_ did not show any antileishmanial activity or cytotoxicity, suggesting that despite having the lowest E_g_ value, it produced minimal ROS under these conditions. This likely resulted from a high recombination rate of electron–hole pairs in Fe^3+^-doped TiO_2_, which in turn reduced the photoactivity of the material.^167^

In a method similar to TiO_2_@Ag, Zn^2+^-doped TiO_2_ was combined with hypericin (HY), a drug approved by US FDA for treating CL.^168^ The authors investigated the photosensitization properties of Zn^2+^-doped TiO_2_, previously demonstrated to enhance photoactivity.^164^ Upon exposure to visible light, this treatment exhibited effective antileishmanial activity against amastigotes forms of *L. amazonensis*, with a IC_50_ of 17.5 μg mL^–1^, and achieving a 58% reduction in parasite burden in infected BALB/C mice, results comparable to those with AmB. However, a low SI of 2.01 suggests that the proposed system lacks specificity for *Leishmania*. Another issue identified was that the addition of HY to the doped NPs did not significantly impact amastigote eradication, possibly due to insufficient macrophage internalization. These findings highlight the need for a thorough investigation into the stability and textural properties of the nanoparticles, which are crucial factors in determining the extent of phagocytosis and cell uptake.

##### Iron Oxide and Magnetic Nanoparticles

Although not their typical use, iron oxide nanoparticles (IONPs) also exhibit semiconductor characteristics, meaning that they can generate ROS under specific conditions. Islam and co-workers synthesized ferromagnetic iron oxide nanorods (FIONs) with median diameter of 70 nm and an adsorption band peak at 262 nm using fenugreek as a reduction, capping, and stabilizing agent.^169^ This innovation was aimed at therapeutic applications against both the promastigote and amastigote forms of *L. tropica* through PDT. For the group exposed to LED light, there was a notable reduction in IC_50_ values from 23.09 and 36.3 μg mL^–1^ to 0.036 and 0.072 μg mL^–1^, for promastigotes and amastigotes, respectively. Thanks to the photocatalytic mechanism and ROS generation, treatment with FIONs proved to be more effective than the positive control (AmB, IC_50_ = 0.55 and 3.4 μg mL^–1^ for promastigote and amastigote forms, respectively). Furthermore, FIONs, which exhibited a high selectivity index (>100), were biocompatible and nonhemolytic, making them promising candidates for local Leishmaniasis treatment via LED irradiation.

IONPs are primarily represented in the magnetic nanoparticles (MNPs) category, mainly consisting of magnetite (Fe_3_O_4_) and maghemite (γ-Fe_2_O_3_) phases.^170,171^ The distinct crystalline structures of these phases primarily determine their magnetic behavior, which stem from vacancies and valence states of iron ions within the sublattices.^172^ Magnetite features a combination of Fe^2+^ and Fe^3+^ ions in a cubic inverse spinel structure.^173^ The ferrimagnetism behavior of magnetite is due to the arrangement of Fe^2+^ ions in octahedral sites and Fe^3+^ ions in both tetrahedral and octahedral sites.^172^ The presence of Fe^2+^ ions means magnetite is readily oxidized in air to form maghemite phase, which retains cubic spinel structure and exhibits ferrimagnetic behavior.^174^

The applications of MNPs in the biomedical field include magnetic resonance imaging (MRI), target drug delivery, and magnetic hyperthermia. Magnetic hyperthermia relies on the movement of the NPs, such as Brownian and Néel relaxations, within physiological media, leading to a localized increase in temperature.^175^ MNPs offer the advantage of controllably generating heat in the presence of an external alternating magnetic field (AMF), thereby avoiding the painful skins lesions that can result from conventional thermotherapy.^45,46,176,177^ Additionally, it is advantageous for MNPs to exhibit superparamagnetic behavior,^45,46,177^ meaning magnetization occurs only when an AMF is applied, and no residual magnetization remains once the AMF is removed.^170,178^ Superparamagnetism is only observed in NPs smaller than 30 nm.^179^

Verçoza and co-workers investigated the ability of superparamagnetic iron oxide nanoparticles (SPIONs, Fe_3_O_4_, 3.8 nm), synthesized using coconut water, to be internalized by macrophages.^180^ They observed no alteration in macrophage morphology and no cytotoxicity at concentrations of up to 300 μg mL^–1^ SPIONs after 24 h of treatment. Small, randomly distributed aggregates were found within the cytoplasm, indicating that SPIONs were internalized through endocytic/phagocytic processes. In the absence of AMF, the IC_50_ for amastigote forms of *L. amazonensis* was 0.67 μg mL^–1^.^181^ Although no assays were performed using AMF, the evidence suggests that SPIONs could represent a promising approach for treating Leishmaniasis.

**Figure 7:**
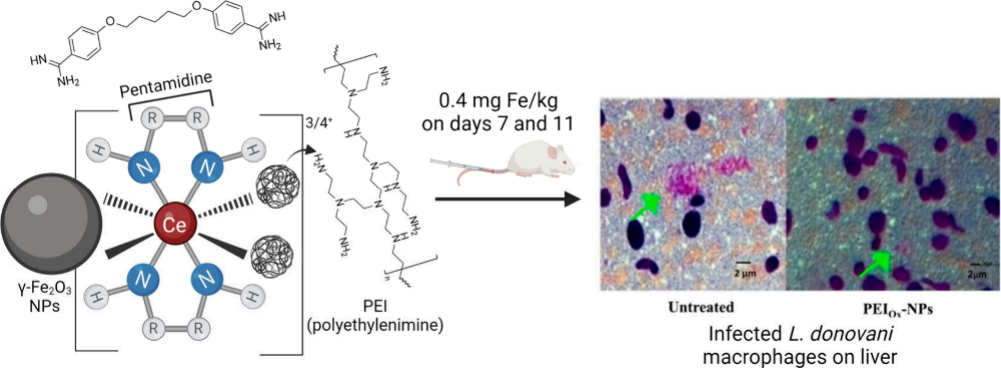
Schematic illustration of the γ-Fe_2_O_3_ NPs functionalized with Ce^3/4+^ complex coordinated by both PEI and pentamidine ligands, developed by Kannan and co-workers. Reproduced from ref (30). Copyright 2021 American Chemical Society.

Kannan and co-workers developed spherical cerium (Ce^3+/4+^)-doped maghemite NPs (CAN-γ-Fe_2_O_3_) sized between 7 and 15 nm and functionalized with polyethylenimine (PEI).^30,182^ The Ce^3+/4+^ served as Lewis’s acid center, facilitating the chelation of PEI through its amino sites. PEI’s “proton sponge” effect, which induces lysosomal rupture by inflowing H_2_O and Cl^–^ anions to lysosome, is particularly lethal to *Leishmania* species that possess only one lysosome.^183,184^ The PEI-decorated CAN-γ-Fe_2_O_3_ exhibited a positive surface charge (+25 to +35 mV), beneficial for electrostatic interactions with the negatively charged protozoan cell surface.^185^ This functionalized system significantly reduced the viability of *L. donovani*, *L. major* and *L. tropica* promastigotes by 90% at concentration of 0.50 μg mL^–1^, outperforming the core CAN-γ-Fe_2_O_3_. It also demonstrated a 90% reduction in the intramacrophage amastigote viability of *L. donovani* at 0.25 μg mL^–1^. A cream formulation of PEI-decorated CAN-γ-Fe_2_O_3_ (0.067% of Fe) showed superior results in treating CL in *L. major*-infected mice compared to the positive control (Leshcutan, 15% paromomycin sulfate and 12% methylbenzethonium). Further, pentamidine was covalently bonded to PEI-decorated CAN-γ-Fe_2_O_3_, showing low macrophage toxicity and high efficacy in reducing parasite forms, indicating a promising treatment option (Figure 7).^182^

Albalawi and co-workers synthesized Fe_3_O_4_ and coated them with piroctone olamine (PO) (Fe_3_O_4_@PO, 15–20 nm).^57^ Piroctone olamine, an antifungal agent used for treating dandruff and fungal infections, has also shown promising apoptotic activity against myeloma.^186,187^ The addition of PO on the Fe_3_O_4_ surface led to in ca. a 50% reduction in the IC_50_ value for inhibiting *L. major* amastigote, from 62.3 μg mL^–1^ (Fe_3_O_4_) to 31.3 μg mL^–1^ (Fe_3_O_4_@PO), a value lower than that of the positive control (MA, 52.6 μg mL^–1^). Although the study did not explore the magnetic properties of the nanoparticles, it was observed that both Fe_3_O_4_ and Fe_3_O_4_@PO induced NO production (20 and 25% NO production at 50 μg mL^–1^, respectively). NO, released by the macrophages, is recognized as a key mediator to parasite elimination.^57^ At a concentration of 100 μg mL^–1^, nearly 100% of amastigotes were inhibited, with no significant toxicity observed in macrophages (CC_50_ > 360 μg mL^–1^). In vivo assays revealed a notable reduction in lesion diameters and parasite burden in BALB/c mice infected with *L. major*, with a 50% reduction in lesion size using Fe_3_O_4_@PO compared to Fe_3_O_4_ alone. Furthermore, a concentration of 2 mg kg^–1^ of Fe_3_O_4_@PO resulted in an 85% reduction in parasite burden, highlighting the potential of Fe_3_O_4_@PO nanoparticles as an effective treatment for Leishmaniasis.

The studies mentioned above demonstrate that Kannan and Albalawi successfully attached drugs to the surface of NPs; however, they did not fully utilize the DDS advantages offered by these nanomaterials such as sustained drug release. Contrarily, Kumar and co-workers developed AmB loaded-Fe_3_O_4_ NPs coated with the amino acid glycine (AmB-GINPs, 10 nm),^29^ employing an amino acid functionalization to enhance system stability and prevent agglomeration while maintaining biocompatibility.^188^ The amino acids interact with magnetite either directly through carboxyl groups or supramolecularly via amino groups, forming hydrogen bonds with hydroxyl groups on the Fe_3_O_4_ surface. This interaction resulted in a zeta potential value of −25 mV at pH = 6.^189^ At the lysosomal pH of infected cells, AmB-GINPs demonstrated a sustained AmB release profile controlled by diffusion, showcasing the system’s effectiveness. The efficacy of this DDS was highlighted by the significantly low IC_50_ values of 4 and 9 ng mL^–1^ for AmB-GINPs and GINPs, respectively, against the amastigote form of *L.donovani*, and a substantial reduction in amastigote burden in infected hamsters, outperforming the positive control.^29^

Although many studies reveal that the antileishmanial effects of oxide nanoparticle-based systems are primarily initiated by the NPs themselves, descriptions of oxide NPs as part of a DDS are scarce in the literature. Iron oxide has shown promising performance in reducing macrophage cytotoxicity and, consequently, selectivity in treating Leishmaniasis with notable in vitro and in vivo efficacy, despite its underexplored magnetic properties. Owing to the ferromagnetic properties of the nanoparticles, these systems could be further investigated in the presence of an alternating magnetic field (AMF), which is expected to enhance nitric oxide (NO) production and, as a result, improve antileishmanial effects through apoptosis and local hyperthermia. Although there is compelling evidence of iron oxide’s potential, other types of metal oxides lack comprehensive data, underscoring the need for further research to meet the requirements for clinical trial advancement.

#### Nonmetallic Oxide Nanoparticles

Regarding nonmetallic oxides, silica nanoparticles (SiO_2_), especially the mesoporous type, have been widely used as platforms for drug delivery systems due to their biocompatibility and versatility, including variable size, and large surface area, and large pore volume.^32,50,190−194^ Furthermore, silica allows for functionalization with various groups that can interact with the silanol moiety present on its surface, facilitating efficient drug loading and cellular uptake.^195^ Thus, by modulating their properties, it is possible to produce a personalized therapy that addresses specific challenges not only for patients but also for reservoir species.^196^ Tsamesidis and co-workers synthesized both mesoporous and nonporous silica doped with Ca^2+^, Mg^2+^ and Cu^2+^ to enhance biocompatibility.^197^ The mesoporous nanoparticles loaded with artemisinin demonstrated improved activity against *L. infantum* amastigotes compared to nonporous doped silica nanoparticles, with a IC_50_ of 1.43 μg mL^–1^. However, due to the absence of toxicity data against macrophages and positive control data, it is difficult to determine the potential of this material as an antileishmanial candidate.

Thapa and co-workers produced mesoporous silica (MSNPs) from barley ash, an agricultural industry residue, and observed a significant in vivo reduction of *L. donovani* parasite burden by incorporating buparvaquone (BPQ) into the MSNPs.^198^ Buparvaquone, a drug with poor water solubility, benefits from improved bioviability when delivered through MSNPs, potentially reducing the toxicity associated with BPQ. The authors found that the BPQ-loaded MSNPs exhibited good biocompatibility, increasing the CC_50_ by ca. 230% compared to pure BPQ (1080 vs 330 μM), and showing 4 and 72-fold increase in CC_50_ compared to PMM and AmB positive controls, respectively. The in vitro results for axenic and intramacrophage amastigotes displayed IC_50_ values similar to those of standard treatments AmBisome and Miltefosine (IC_50_ ∼ 1.0 μM against ∼0.3 and 0.5 μM, respectively) and lower than those for BPQ, sodium stibogluconate (SSG) and paromomycin (PMM). In vivo studies on BALB c/mice demonstrated a 98% reduction in spleen parasite burden with BPQ-loaded MSNPs, compared to a 64% reduction with pure BPQ, significantly enhancing the drug’s antileishmanial effect while maintaining safe levels of biological toxicity markers in the liver and kidney. These findings suggest that the reported system is a promising candidate for clinical trials against VL, with the potential to address the toxicity issues associated with current market drugs while remaining effective.

### Carbon-Based Materials

Carbon-based materials consist of both natural forms, e.g., graphite and diamond, and synthetic ones, including fullerenes, carbon nanotubes, and graphene. These materials can form various allotropes due to the close energy of the *2s* and *2p* orbitals, which enables the formation of hybridized orbitals. The differences among these allotropes lie in their spatial configurations, type of carbon atoms hybridization and, consequently, their properties.^199^ Their applications in the biomedical field are closely related to their spectroscopic characteristics.

Fullerenes are composed of a spherical arrangement of 60 *sp*^2^ carbons, organized into 12 pentagons and 20 hexagons, resembling a soccer ball.^200,201^ They can absorb photons in the UV–vis region, generating ROS, and thus can be utilized in photodynamic therapy for cancer treatment, as blood sterilant, and in cosmetic formulations.^202^ Carbon nanotubes are formed from sheets of graphite rolled into a cylindrical shape.^203^ Their optical, electrical and mechanical properties vary depending on the number of layers, with notable characteristics including high thermal and electrical conductivity–surpassing metallic conductors–and high mechanical resistance. These properties make carbon nanotubes suitable for applications in bone and cartilage regeneration.^202,204^ Lastly, graphene is a single layer of graphite consisting of a monolayer of *sp*^2^ carbon atoms arranged in a honeycomb-like pattern. Its high surface area enables the adsorption of many molecules, making it an efficient drug carrier.^205^ Among its advantages, the most significant for biomedical applications is its potential photothermal effect, stemming from the ability of the conjugated C=C bonds to absorb radiation in the near-infrared region (NIR).^206^ Until now, obtaining these carbon-based materials with high quality and on a large scale has been costly, and environmentally unfriendly, contradicting the essence of the One Health concept. One solution is to produce them from biomass waste, which addresses both cost and scalability issues. Furthermore, developing sustainable and efficient carbon-based materials through waste reduction can be an excellent option, promoting environmental sustainability while advancing technological progress.^207^

Sundar’s research group has a history of investigating carbon-based materials for the treatment of Leishmaniasis. In their inaugural publication in 2011,^59^ they described an amino-functionalized carbon nanotube (f-CNT) and its efficacy against *L. donovani*. The amino functionality not only increased the solubility of CNT in an aqueous environment but also served as an anchor for attaching amphotericin B onto CNT structure through covalent bond, resulting in a material named f-CNT-AmB. Cytotoxicity results indicated that f-CNT was less toxic to macrophages than pure AmB, with CC_50_ values of 7.31 and 0.48 μg mL^–1^, respectively. Although the cytotoxicity of f-CNT-AmB was similar to that of pure AmB, its activity against intramacrophage amastigotes (IC_50_ = 0.0023 μg mL^–1^) was almost 14 times more effective than that of AmB (IC_50_ = 0.033 μg mL^–1^). Thus, with a higher selective index, the administration of f-CNT-AmB presents a promising alternative to conventional treatments. In vivo assays yielded encouraging results. The toxicity of AmB, f-CNT and f-CNT-AmB was first examined in BALB/c mice by administering varying concentrations (5, 10, and 20 mg kg^–1^ per day) for 5 days through intraperitoneal injections. Although an inflammatory response was observed at the injection site, no renal or hepatic toxicity was reported for any concentrations. In vivo antileishmanial assay in infected hamsters treated with AmB, f-CNT and f-CNT-AmB (5 mg kg^–1^ per day) revealed that f-CNT-AmB inhibited splenic parasite load by almost 90%, compared to 45% with pure f-CNT and 69% with AmB. This effect was also reflected in spleen weight, which decreased from 0.9 g (before treatment) to 0.8 g (AmB) and 0.6 g (f-CNT-AmB), as opposed to an increase for the control group (1.5 g) and the f-CNT-treated group (1.1 g). In subsequent research,^58^ the group explored oral administration of the same material. They prepared a formulation of f-CNT-AmB in saline phosphate buffer (PBS) at concentrations 1, 2, and 4 mg mL^–1^ and administrated it both orally and intraperitoneally. For comparison, miltefosine and AmBisome were used for oral and intraperitoneal administration, respectively. The results showed no significant differences between the two methods at the same concentration, and f-CNT-AmB demonstrated similar effectiveness to miltefosine, encouraging further investigation of these materials. Following the same strategy, in 2014, they investigated the potential antileishmanial activity of betulin-functionalized carbon nanotubes (f-CNT-BET).^60^ Betulin (BET), a pentacyclic triterpenoid molecule that inhibits the trypanothione synthetase, an important redox enzyme in parasite homeostasis, was attached to the f-CNT surface via carbodiimide-based esterification reaction.^208,209^ Despite concerns that covalent bond between BET and f-CNT could hinder drug action, BET release studies demonstrated that the bond underwent hydrolysis in an acid environment, resulting in releases of 12.5 and 38.4% at pH values of 7.4 and 5.8, respectively. Notably, the study reported no significant cytotoxicity, especially when compared to the IC_50_ values for intramacrophage amastigotes.

Sundar’s group transitioned from using carbon nanotubes (CNT) to graphene oxide (Gr) because the production process for Gr is simpler and more scalable than that for CNT.^61,210^ Additionally, graphene sheets offer a theoretical surface area of ca. 2630 m^2^ g^–1^,^211,212^ which can enhance drug loading capacity. In this work, they employed the same approach of functionalizing with amino groups to improve aqueous dispersion, followed by attachment of AmB to the f-Gr. The preliminary results were promising, showing an antiproliferative effect against intramacrophage amastigotes similar to that of f-CNT-AmB. In vivo studies demonstrated an 88% inhibition of splenic parasite load and a 90% suppression of parasite replication, compared to 70 and 76%, respectively, for pure AmB. Interestingly, the f-Gr without AmB attached did not exhibit significant antileishmanial activity.

Recently, Sundar’s group reported on a composite formed by functionalized Gr and CNT with AmB.^62^ In vitro and in vivo assays were conducted following the methods described in their earlier works.^58,59,61^ The in vivo studies demonstrated a slight improvement in the inhibition of splenic parasite load (96%) and suppression of parasite replication (98%) in those treated with the loaded composite, named f-Comp-AmB. The authors proposed that this enhanced effect resulted from a synergy between Gr and CNT.

Although Sundar’s group findings are promising, the high doses required could pose limitations for further clinical trials. This issue may be attributed to the covalent bond between AmB and the carbon-based materials, potentially diminishing the drug’s effectiveness. As an alternative, several researches are exploring the incorporation of drugs into carbon system through weaker interactions, such as hydrogen bond and van der Waals, to act as efficient DDS.^213^ This approach aims to facilitate the targeted delivery and on-demand release of the drug to infected cells. Contributing to this discussion, Ronconi and co-workers synthesized two drug delivery systems based on reduced graphene oxide (rGO) and biocompatible polymers.^185,214^ The reduction of graphene oxide restores conjugated π bonds, enabling the material to absorb NIR radiation and convert it into localized heat, suggesting the potential for photothermal applications. However, to overcome rGO’s lack of biocompatibility, the team functionalized it with two different polymers: Pluronic P123 (P123) and polyethylenimine (PEI), resulting in rGO-P123 and rGO-PEI, respectively. The photothermal effect was investigated by irradiating material dispersions at pH = 7.4 (physiological) and 5.0 (intramacrophages) with an infrared lamp, leading to temperature increases of 8.2 and 8.7 °C in an acidic environment for rGO-P123 and rGO-PEI, respectively. Both materials were loaded with AmB to assess the antileishmanial activity and cytotoxicity. Kinetic studies of AmB release revealed that, under NIR radiation, rGO-PEI material released AmB at rates of 4.72 and 6.70 μg mL^–1^ at pH = 7.4 and 5, respectively, - twice the amount released in the absence of radiation. For rGO-P123, the presence of NIR radiation did not significantly affect the amount of AmB released. The higher drug release from rGO-PEI, compared to rGO-P123, was attributed to PEI’s reducing properties, making rGO-PEI more reduced and thereby more responsive to NIR radiation.^185,214^

The evaluation of chemical characteristics is crucial as they dictate the biological properties of the materials, a key focus of Ronconi’s work. Using different polymers yielded materials with varying surface charges: rGO-P123 exhibited a negative surface charge, and rGO-PEI had a positive one. Cell viability studies on macrophage cell lines revealed that compared to the control, the rGO-P123 system was toxic to macrophages, whereas the rGO-PEI system did not cause any harm. Consequently, the antiproliferative effect of rGO-PEI, both with and without the drug, against *L. amazonensis* was assessed. Given that the parasite’s cell wall is composed of negatively charged glycophospholipids, the interaction of the positively charged rGO-PEI system loaded with AmB, especially in the presence of NIR radiation, resulted in approximately 80% growth reduction. In contrast, control tests with only AmB at equivalent concentrations achieved a mere 11% growth inhibition, underscoring the potential of the positively charged system in treating Leishmaniasis.^185,214^

Similarly, Singh and co-workers explored the use of epoxy-targeted selectively functionalized GO with ethylenediamine conjugation (AGO) as a carrier for AmB.^215^ The drug was loaded onto GO and AGO surfaces through hydrogen bonds among several functional groups. The introduction of amino groups enhanced the potential interactions between AmB molecules and the materials, as evidenced by the loading efficiencies of 43 and 55% for GO-AmB and AGO-AmB, respectively. Amino functionalization significantly influenced the surface charge of the material, shifting from −26 (GO) to +9 mV (AGO). After loading AmB, the surface charges changed to −35 mV (GO-AmB) and −7 mV (AGO-AmB). Despite GO having a larger surface charge in absolute value, which suggests better colloidal dispersion, in vitro studies on cellular uptake showed a decrease in the uptake of GO-AmB compared with AGO-AmB. This was attributed to the negative surface charge of the parasite cell walls, which repelled the negatively charged GO-AmB surface. Cytotoxicity results indicated that after 24 h of treatment, both unloaded and AmB-loaded materials did not exhibit significant cytotoxicity, maintaining cell viability above 80%, compared to 67% for pure AmB (CC_50_ values are not given). Moreover, the IC_50_ values against *L. donovani* amastigotes of GO-AmB and AGO-AmB were 5 and 2 times lower than that of pure AmB, respectively, demonstrating their antileishmanial activity.

Interestingly, Ramos and co-workers investigated the antileishmanial effect of hydroxyl-functionalized fullerenes, known as fullerol or fullerenol (Ful).^216^ While fullerenes are recognized for their anti-inflammatory and antioxidant properties, they exhibit low water solubility. Chemically modifying fullerenes with hydroxyl groups enhances their solubility without compromising their antioxidant activity.^217^ In their study, Ful was not used as a nanocarrier but rather acted as the drug itself, encapsulated with a mixture of two polymers distearoylphosphatidylcholine (DSPC), dipalmitoylphosphatidylglycerol (DPPG) and cholesterol. Two formulations were prepared using the dehydration–rehydration method, with different amount of Ful (15 or 60 μg mL^–1^, resulting in Lip-Ful1 and Lip-Ful2, respectively). Additionally, a third formulation was created using a sucrose solution to simplify the preparation process (Lip-Ful2-suc). The encapsulation efficiency for Lip-Ful2 was quantified at 25%. In vitro studies showed that Ful had an IC_50_ value of 42 μg mL^–1^ against intramacrophage *L. amazonensis* amastigotes, which is twice the concentration needed compared to glucantime (IC_50_ = 20 μg mL^–1^), for the same model over 72 h. However, Ful exhibited no significant cytotoxicity at concentrations up to 120 μg mL^–1^. For in vivo assays, two models were used: one against *L. amazonensis* for acute VL and another against *L. infantum* for chronic VL, resulting in three total in vivo assays. The *L. amazonensis* assay evaluated the efficacy of free Ful daily for 20 days, starting 7 days postinfection in BALB/c mice at a concentration of 0.05 mg kg^–1^, compared to control and glucantime (120 mg Sb(V) kg^–1^). The liposomal formulations (Lip-Ful) were then tested with dosages administered every 4 days, with concentrations of free Ful and Lip-Ful formulations increased 4-fold (0.2 mg kg^–1^ every 4 days) to maintain the same total dosage. This latter treatment began two months postinoculation with *L. infantum* promastigotes and lasted 24 days, using the same dosage regime but comparing it to miltefosine (10 mg kg^–1^ daily) as the positive control. In the first in vivo assay, Ful matched glucantime’s ability to reduce the liver parasite burden but was less effective in the spleen. Therefore, liposomal formulations were employed in the second in vivo assay to achieve comparable results in both organs. The Lip-Ful formulations reduced the parasite burden in both the liver and spleen, with Lip-Ful2 achieving complete eradication of liver parasites. The work by Ramos and colleagues suggests potential for further exploration of this class of materials as nanocarriers for various drugs, such as miltefosine and amphotericin B.

## CLINICAL TRIALS AND ECONOMIC FEASIBILITY: WHERE ARE WE?

**Figure 8:**
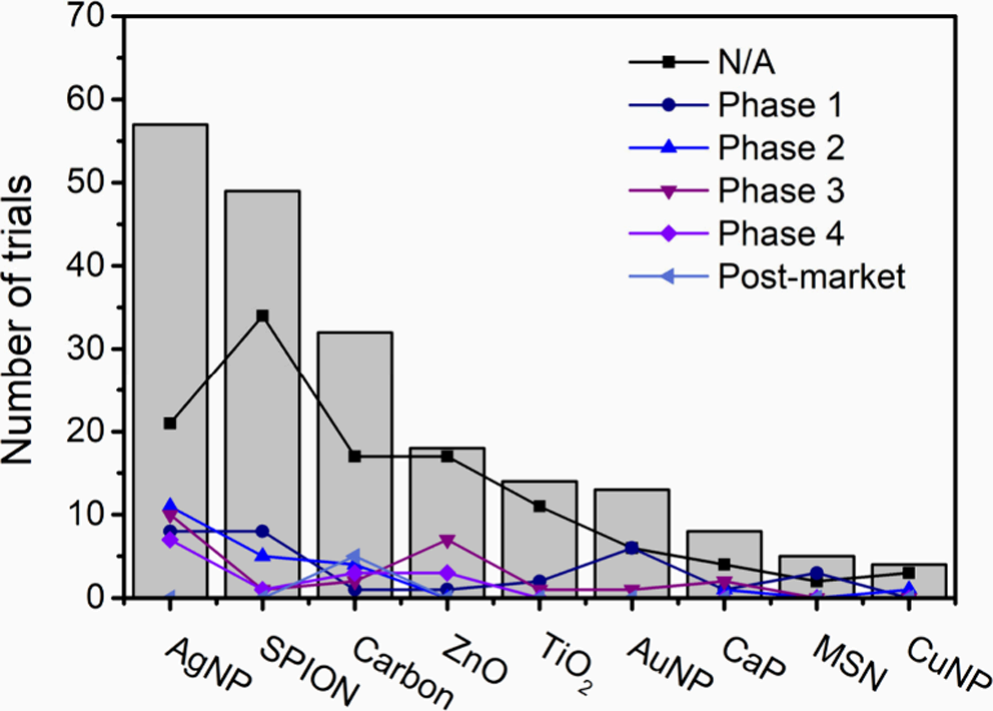
Number of trials using inorganic nanoparticles according to the type of NPs and their current clinical phase.

Even though there are several research papers investigating inorganic nanoparticles for the treatment of not only Leishmaniasis but also cancer^218^ and microbial infections,^219^ there is still a long way to go. A search conducted through the International Clinical Trials Registry Platform by WHO^220^ using the keyword “nanoparticles” returned 341 results in which 200 trials were related to inorganic nanoparticles, highlighting AgNPs, SPIONs, carbon NPs, ZnO, TiO_2_, AuNPs, calcium phosphates (CaP), MSN and CuNPs (Figure 8).

From these data, there are some interesting features to discuss. First, inorganic nanomaterials are not the minority among the clinical trials, representing ca. 60% of the results. However, among the 200 trials, only one is related to Leishmaniasis (NCT06000514) conducted by the Hospital Universitário Professor Edgard Santos (BA, Brazil). In this study, the adverse reactions and the best dose of topical application of Sm29 protein on the surface of AuNPs combined with intravenous MA in the treatment of CL caused by *L. Braziliensis.* Sm29 protein is an antigen present on the *Schistosoma mansoni* adult worm tegument surface, main agent of schistosomiasis, and it is related to a T-helper (Th)1 inflammatory response.^221^ In the case of Leishmaniasis, Th1 response is essential to the infection control.^222^ Therefore, the study was performed to compare the efficacy of MA associated with Sm29, with meglumine antimoniate plus placebo and meglumine antimoniate alone in the treatment of CL. However, to the best of our knowledge, no result nor research paper regarding this investigation was published. An interesting approach that could be applied to CL treatment is the topical administration of NPs. A phase 4 trial evaluated the efficacy of AgNPs hydrogels compared with a reference hydrogel for wound management (TCTR20230623001). The results showed that both treatments had similar effect in wound area reduction and pain score but AgNP-based hydrogels had better result in preventing bacterial infections for the period of 14 to 21 days.^223^

Comparing the current clinical trials to the number of research papers (over 1 million as of May 2024 using the keyword “inorganic nanoparticles” in the SciFinder database), there is still much we do not know. Specifically, the lack of thorough understanding of nanobio interactions and toxicity are the most significant factors that block the way through clinical translation.^53^ Nowadays, toxicity tests with fishes, e.g., *Danio rerio* zebrafish, can provide great information regarding the fate of NPs inside the body and possible environmental effects.^224−226^ Additionally, the advancement of tissue engineering^227^ and artificial intelligence^228^ can help rationalize the design of new materials based on the challenges faced by advanced studies.

In terms of cost, large-scale production involving specialized equipment, high-purity chemicals, and complex manufacturing processes are the primary challenges in translating inorganic nanoparticles to clinical applications. Ensuring uniformity, purity, and stability through sophisticated characterization methods and rigorous quality testing also adds to production costs.^229^ However, preclinical and clinical trials, essential for safety and efficacy evaluation, and regulatory approval processes are expensive and time-consuming, leading to limited investment in early stage development due to high risks and uncertain financial returns further hindering progress.^53^

## CONCLUSION AND OUTLOOK

In conclusion, our literature review emphasizes the promising yet underexplored role of inorganic nanomaterials in treating Leishmaniasis. The variety of nanomaterials discussed, including metallic nanoparticles, oxides, and carbon-based materials, highlights their potential as effective therapeutic agents against *Leishmania*. The unique physicochemical properties of these nanomaterials, such as chemical composition, size, morphology, and surface charge, play a crucial role in their antileishmanial activity by facilitating membrane penetration and inducing cell death. However, further exploration of synthesis methods to modify their compositions and surfaces could lead to more efficient systems. For instance, the challenge of minimizing harm to macrophages could be addressed by surface functionalizing nanomaterials with biocompatible molecules, thereby increasing the selectivity index essential for optimal treatment.

Throughout this review, we observed that inorganic metallic nanoparticles react to pH changes by undergoing oxidation and generating reactive oxygen species (ROS). Metallic oxide nanoparticles leverage their unique optical properties to induce ROS production through light irradiation, and their magnetic properties can be exploited for inducing hyperthermia or drug release in drug delivery systems (DDS). Non-oxide nanoparticles, such as mesoporous silica nanoparticles, have been extensively researched as reservoirs for sustained drug release, showcasing significant potential not yet fully applied to Leishmaniasis. Similarly, carbon-based materials have yielded notable results against *Leishmania* strains, albeit with limited studies. Graphene-based materials’ ability to absorb near-infrared (NIR) light and fullerenes’ capacity to absorb UV light can be harnessed to trigger ROS production, akin to semiconductor nanoparticles.

Despite promising in vitro and in vivo outcomes, ongoing challenges related to biocompatibility, toxicity, and clinical translation must be addressed. By continuing to integrate these nanomaterials into treatment modalities, we could significantly enhance both the efficacy and the specificity of interventions against Leishmaniasis. This effort would not only address a critical public health issue but also align with the One Health concept, promoting an integrated approach to health that considers human, animal, and environmental well-being.

## Abbreviations

47-DHF: 4′,7-dihydroxyflavone
AgNPs: silver nanoparticles
AMF: alternating magnetic field
AmB: Amphotericin B
AuNP: gold nanoparticles
AuNRs: gold nanorods
BET: betulin
BNP: bimetallic nanoparticles
BPQ: buparvaquone
CaP: calcium phosphates
CC_50_: half maximal cytotoxicity concentration
CL: cutaneous Leishmaniasis
CNT: carbon nanotube
CuNPs: copper nanoparticles
DDS: drug delivery systems
DPPG: dipalmitoylphosphatidylglycerol
DSPC: distearoylphosphatidylcholine
FDA: Food and Drug Administration
FIONs: ferromagnetic iron oxide nanorods
Ful: fullerol or fullerenol
GA: gallic acid
Gr: graphene oxide
GRASE: generally recognized as safe and effective
HY: hypericin
IC_50_: half maximal inhibitory concentration
IONPs: iron oxide nanoparticles
LED: light emitting diode
LP: lipoic acid
MA: meglumine antimoniate
MO-NPs: metallic oxide nanoparticles
MNPs: magnetic nanoparticles
MSNPs: mesoporous silica nanoparticles
MRI: magnetic resonance imaging
NIR: near infrared region
NPs: nanoparticles
NS: Nigella Sativa oil
NTD: Neglected Tropical Diseases
NW: New World
OW: Old World
P123: Pluronic P123
PDT: photodynamic therapy
PEG: polyethylene glycol
PEI: polyethylenimine
PLGA: poly(lactic-*co*-glycolic acid)
PMM: paromomycin
PO: piroctone olamine
PVP: polyvinylpyrrolidone
rGO: reduced graphene oxide
ROS: reactive oxygen species
SDGs: Sustainable Development Goals
SEM: scanning electronic microscopy
SERS: surface-enhanced Raman spectroscopy
SI: selective index
SPIONs: superparamagnetic iron oxide nanoparticles
SSG: sodium antimony gluconate
TEM: transmission electronic microscopy
VL: visceral Leishmaniasis
WHO: World Health Organization
XRD: X-ray diffraction
